# Correction: A SCARECROW-RETINOBLASTOMA Protein Network Controls Protective Quiescence in the Arabidopsis Root Stem Cell Organizer

**DOI:** 10.1371/journal.pbio.1001997

**Published:** 2014-10-20

**Authors:** The PLOS Biology Staff

Since publication of this paper, the authors became aware of several incorrect or missing details that required correcting. The authors have provided corrected versions, along with explanations, here.

1. Figure 1A-D have been used again in Figures S2B, S2F and S2H. This was deliberate, and on request of a referee for more detail, but we did not explicitly mention it in the figure legend. The corrected figure legend for Figure S2 has been provided here.
2. Figure 2E: an RT-PCR experiment is shown in which overexpression of an artificial microRNA against RBR (amigo-RBR) reduces RBR RNA levels. We used two different promoters for overexpression, pG1090 XVE and 35S, and both yielded exactly the same result. We inadvertently used results for pG1090 XVE and named them 35S::amiGO-RBR in the original figure, and have now replaced these with the results for 35S::amiGO-RBR. In addition, because samples were run in separate slots on the same gel, we put a white horizontal line between the RBR and ACTIN bands. The corrected Figure 2 file has been provided here.
3. Figure 3M uses an image that was shown again in Figure S1J for different features. We now indicate this explicitly in corrected Figure 3 and Figure S1 legends, which are shown here.
4. Figure 3J and Figure 3T inadvertently contained images that we had previously published elsewhere, focusing on different aspects of the phenotype that emerged from the analysis of the same set of data. In the corrected Figure 3, provided here, we have replaced these panels with other representative images from the same data set which are not used elsewhere. The replicate data and choice of appropriate alternate panels for this correction was overseen by our institution.

**Figure 2:**
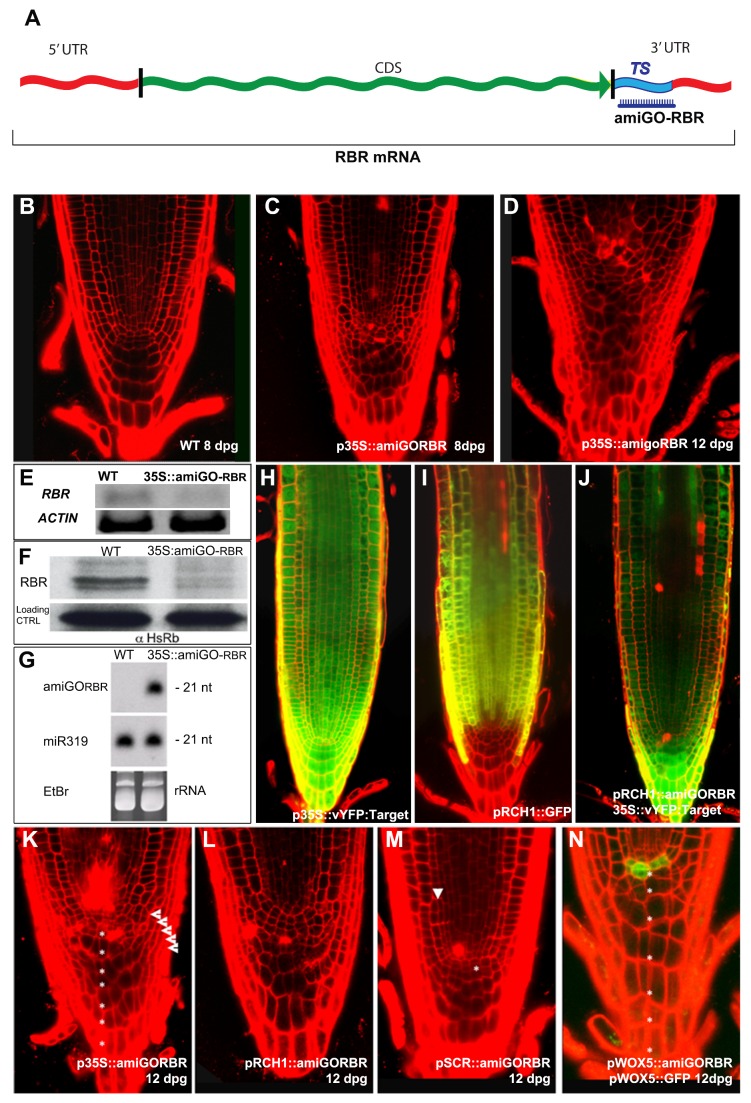
The AmiGO concept for RBR silencing. The AmiGO strategy. TS, Target sequence (A). Root apical meristem (RAM) of WT (B), *35S:amiGORBR* at 8 (C) and 12 (D) dpg seedlings. Validation of the amiGORBR RNA interference by RT-PCR detection of endogenous RBR transcripts (E) and Western blot analyses for RBR protein levels (F). Mature amiGO-RBR synthesis detected by small-RNA Northern blot (G). The *in planta* action of amiGO-RBR was revealed by introducing the sensor construct *35S::vYFP:amiGORBR-TS* in WT (H) and in the *pRCH1::amiGORBR* (J) backgrounds. Expression pattern of the *pRCH1::GFP* marker in the WT background (I) [54]. AmiGORBR expression driven by different promoters generates distinct phenotypes in primary root meristems of 12 dpg seedlings. (K) *p35S::amiGORBR* causes overproliferation of QC, LRC (arrow heads), and CSC (asterisks) as well as cell death in vascular and columella cells. (L) *pRCH1::amiGORBR* shows overproliferation of the QC and LRC and cell death and (M) *pSCR::amiGORBR* shows extra periclinal divisions of the ground tissue (arrow heads) and extra division of the QC (asterisks), while (N) *pWOX5::amiGORBR;pWOX5::GFP* shows QC divisions that cause an increase in columella layers (asterisks). See also Figures S3, S4, S5.

**Figure 3:**
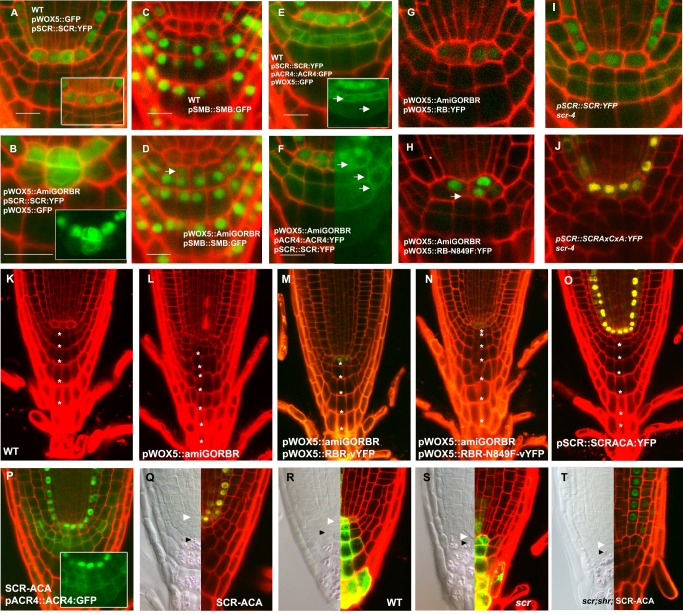
RBR silencing induces asymmetric cell division of the QC. Expression patterns of cell-fate markers in stem cell niche of WT (A, C, and E) and *pWOX5:amiGORBR* (B, D, and F) 5 dpg seedlings. Marker genotypes: cytoplasmic *pWOX5:GFP* and nuclear *pSCR:SCR:YFP* (A and B), *pSMB::SMB:GFP* (C and D), cytoplasmic *pWOX5:GFP*; nuclear *pSCR:SCR:YFP* and membrane *pACR4:ACR4:GFP* (E and F). Arrows in (D) indicate shift in SMB expression, and arrows in (E) and (F) point to plasma membrane-localized ACR4-GFP expression. Full rescue of the QC division in *pWOX5:amiGORBR* complemented with *pWOX5:RBR:YFP* and scr-4 complemented with *pSCR::SCR:YFP* (G, I) and no rescue by LxCxE mutant *pWOX5:RBRN849F-YFP* or *pSCR::SCRAxCxA:YFP* (H, J). Root phenotype of 12 dpg seedlings of Col-0 WT (K), *pWOX5::amiGORBR* (L), *pWOX5::amiGORBR;pWOX5::RBR:vYFP* (M), *pWOX5::amiGORBR;pRBR::RBRN849A:vYFP* (N), and *pSCR::SCRAxCxA:YFP, scr-4* (O). Asterisks depict the columellla layers rootwards from the layer in contact with the QC and excluding the detaching distal layers. Production of extra columella stem cell in *pSCR::SCRAxCxA:YFP, scr-4* as shown with ACR4-GFP marker (P). Number of columella stem cell layers by lugol staining in *pSCR::SCRAxCxA:YFP, scr-4* (Q), Col-0 WT (R), *scr-4* (S), and *pSCR::SCRAxCxA:YFP shr-2 scr-4* plants (T). See also Figure S6 and Figure S1, where Figure 3M is redisplayed for QC identification.

None of these changes affect in any way the results and conclusions reported in the paper.

## Supporting Information

Figure S1 **Root meristem in *Arabidopsis thaliana*.** Different cell types in the root apical meristem of *Arabidopsis thaliana*. Quiescent center, QC; Columella Stem Cell, CSC; Columella differentiated, Col; Lateral Root Cap, LRC; Epidermis, Epi; Cortex, Cor; Endodermis, En; Vasculature, Vasc. Cortex and Endodermis comprise the ground tissue (green); the columella tissue is represented in orange, and QC cells are yellow. (A) SCR expression domain, in QC, ground tissue stem cells and endodermis (B), and WOX5 expression domain in QC of the complemented *pWOX5::RBR:YFP pWOX5::amiGORBR* line, shown in Fig 3M (C).
(TIF)

Figure S2 **QC incorporates F-ara-EdU at longer times than surrounding stem cells**. Left images show red (F-*ara*-EdU) and blue (DAPI staining) channels; right pictures show overlayed green (*pSCR::SCR:GFP*) channel using images shown in Figure 1 A-D. Arrowhead shows QC region that is stained by *pSCR::SCR:GFP*. Note that all green nuclei have no F-*ara*-EdU signal at 1–3 dat, but they show signal at 4 dat. Root meristem shown (A–B) 1 dat, (C–D) 2 dat, (E–F) 3 dat, and (G–H) 4 dat.
(TIF)
