# Supplementary figures and images for: Correction: A SCARECROW-RETINOBLASTOMA Protein Network Controls Protective Quiescence in the Arabidopsis Root Stem Cell Organizer
Source: PLoS Biol. 2014 Oct 20;12(10):e1001997. doi: 10.1371/journal.pbio.1001997 (PMC4203484; doi:10.1371/journal.pbio.1001997)

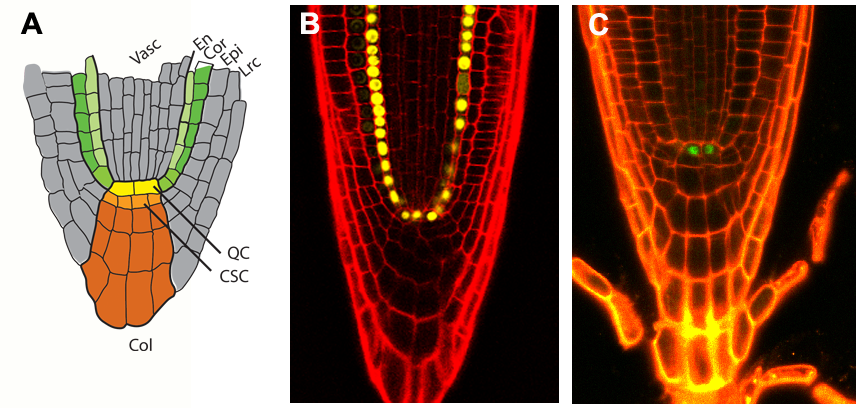

Supplement: Figure S1 — Root meristem in Arabidopsis thaliana. Different cell types in the root apical meristem of Arabidopsis thaliana. Quiescent center, QC; Columella Stem Cell, CSC; Columella differentiated, Col; Lateral Root Cap, LRC; Epidermis, Epi; Cortex, Cor; Endodermis, En; Vasculature, Vasc. Cortex and Endodermis comprise the ground tissue (green); the columella tissue is represented in orange, and QC cells are yellow. (A) SCR expression domain, in QC, ground tissue stem cells and endodermis (B), and WOX5 expression domain in QC of the complemented pWOX5::RBR:YFP pWOX5::amiGORBR line, shown in Fig 3M (C). (TIF) [file pbio.1001997.s001.tif]

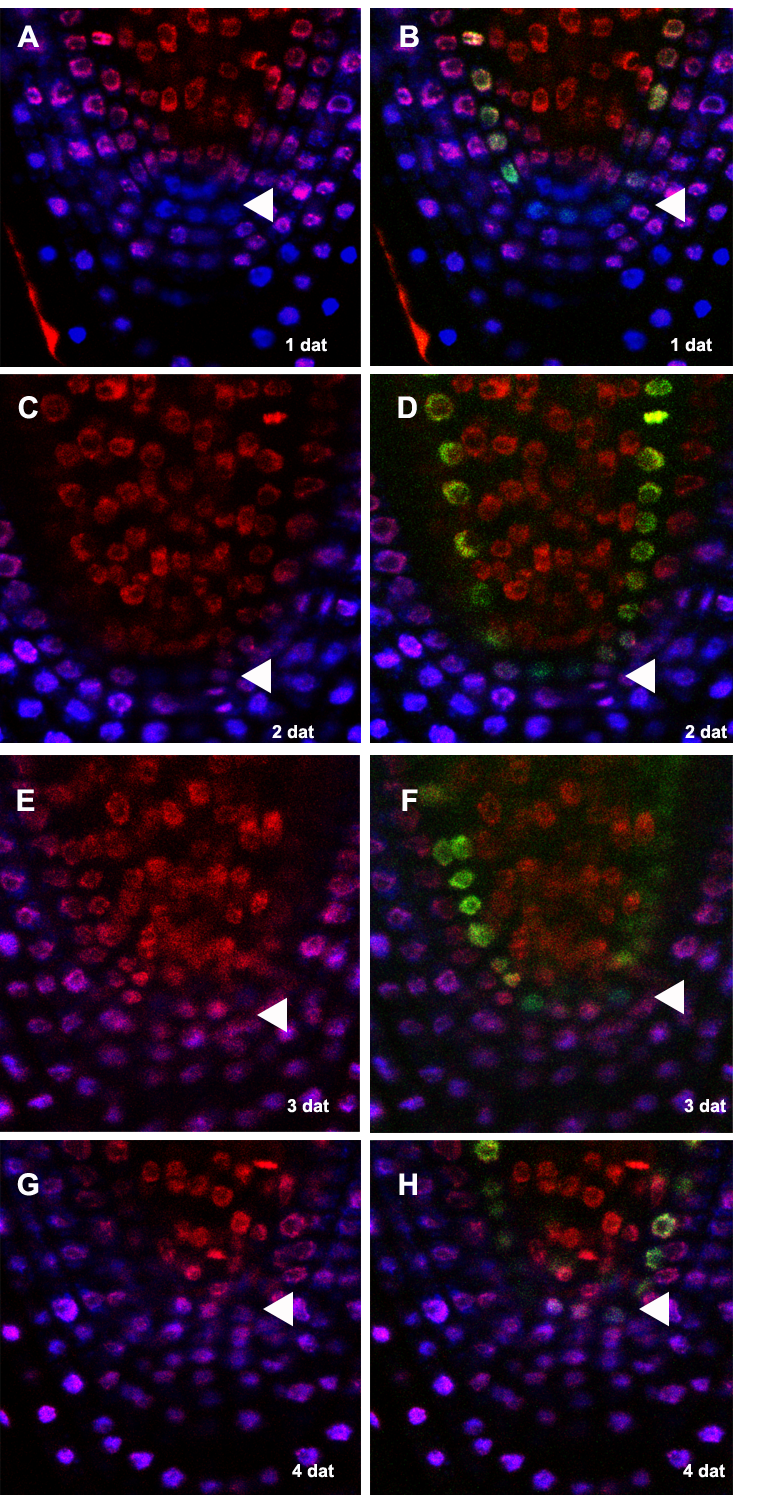

Supplement: Figure S2 — QC incorporates F-ara-EdU at longer times than surrounding stem cells. Left images show red (F-ara-EdU) and blue (DAPI staining) channels; right pictures show overlayed green (pSCR::SCR:GFP) channel using images shown in Figure 1 A-D. Arrowhead shows QC region that is stained by pSCR::SCR:GFP. Note that all green nuclei have no F-ara-EdU signal at 1–3 dat, but they show signal at 4 dat. Root meristem shown (A–B) 1 dat, (C–D) 2 dat, (E–F) 3 dat, and (G–H) 4 dat. (TIF) [file pbio.1001997.s002.tif]
